# Exploring the complex mechanisms of post-intracerebral hemorrhage depression: towards personalized treatment approaches

**DOI:** 10.3389/fpsyt.2025.1651207

**Published:** 2025-10-22

**Authors:** Pengpeng Li, Yangyang Gao

**Affiliations:** ^1^ Department of Neurosugery, Xi’an Aerospace Hospital of Northwest University, Xi’an, Shaanxi, China; ^2^ Ningxia Medical University, Yinchuan, China

**Keywords:** post-stroke depression (PSD), intracerebral hemorrhage (ICH), neuroinflammation, neurotransmitter dysregulation, neurobiological mechanisms

## Abstract

Post - stroke depression (PSD) is a common mental disorder after stroke that significantly impacts patients’ quality of life. While research on depression after ischemic stroke has made progress, the mechanisms of depression after cerebral hemorrhage remain unclear. The incidence of depression after cerebral hemorrhage is high, ranging from 18% to 60%, which greatly affects patients’ rehabilitation and quality of life. This article reviews the pathogenesis, clinical manifestations, and treatment approaches for depression following intracerebral hemorrhage (ICH), emphasizing its distinct characteristics and therapeutic challenges, while also outlining potential directions for future research. The development of depression after ICH is multifactorial and complex. Firstly, disruptions in neurotransmitter systems may be a critical underlying mechanism. Secondly, neuroinflammatory processes likely contribute to its onset. Additionally, the interplay between neural network reorganization and psychosocial factors must also be considered. Current treatments for depression after a brain bleed include medication, non-drug therapies, and teamwork among healthcare professionals. Medications can help balance brain chemicals to reduce symptoms. Non-drug therapies, like counseling and support groups, offer emotional help and ways to manage stress. Working together, doctors, therapists, and other experts create personalized plans to improve recovery. Future research should focus on combining precision medicine and new technologies to improve personalized treatment and practical use for depression after ICH. Precision medicine can customize care based on a patient’s unique traits, such as genetic data and biological markers. Advances in brain imaging and genetic testing can help us better understand the causes of this condition and provide more effective and tailored treatments.

## 1 Introduction

ICH, recognized as the most severe form of stroke, is characterized by high morbidity and substantial disability rates (1). Primary ICH is usually caused by an underlying condition called cerebral small vessel disease (CSVD). This is a long-term issue with the brain’s small blood vessels, which leads to a buildup of damage in the brain over time, including both strokes and bleeds (2). Compared to survivors of other stroke types, ICH survivors exhibit higher risks of recurrent stroke and accelerated cognitive decline (3).

ICH is a common type of stroke that still presents major challenges, even with modern medical advancements (4). While better emergency care has improved survival, long-term issues—especially mental health problems—remain a key area of study (5). Depression after ICH (PICH-D) is the most frequent mental health complication, affecting 18-60% of survivors and significantly impacting their recovery and daily life (6). Distinct from post-stroke depression following other stroke types, PICH-D pathogenesis is closely associated with unique pathophysiological mechanisms of cerebral hemorrhage, including hematoma mass effect, neuroinflammatory responses, and iron metabolism dysregulation (7).

The link between ICH and depression can be explained through three main pathways. First, the blood clot presses on nearby brain tissue, disrupting how brain cells communicate and affecting signals related to emotions (8). This damage to the brain’s structure, especially in areas that control emotions, may increase the risk of depression (9).

Second, When the brain experiences bleeding, the initial inflammation is a protective reaction. However, if this inflammation continues for too long or becomes too intense, it can lead to harmful effects (10). Over time, this ongoing inflammation not only increases the death of brain cells but also disrupts the balance of chemicals in the brain. This creates a foundation for mood disorders like depression or anxiety (11).

Third, the disruption of iron homeostasis following erythrocyte lysis leads to pathological iron deposition (12). This excess iron can trigger harmful chemical reactions, producing toxic molecules that damage brain cells and their energy centers. This damage, especially in areas of the brain that control mood, can worsen and contribute to the development of depression (13).

In recent years, medical research has made steady progress in understanding brain and mental health disorders (14). Scientists have made important strides in uncovering the causes of depression that can follow ICH. At the same time, treatments for this condition have improved significantly, becoming more targeted and varied.

This article provides a detailed review of research on depression after cerebral hemorrhage. It explains how ICH can lead to depression, looking at factors like the pressure from the bleed, brain inflammation, and problems with iron in the body. It also discusses treatment options, including medications, non-drug therapies, and teamwork among healthcare providers. Lastly, it suggests areas for future research to improve how we diagnose and treat this condition. The goal of this article is to be a useful resource for doctors and researchers.

## 2 Pathogenesis: a complex network of multifactorial interactions

PICH-D is caused by a mix of many factors working together (15). These include changes in brain chemistry, inflammation in the brain, rewiring of brain networks, and emotional or social stress (16).

For example, imbalances in brain chemicals like dopamine, norepinephrine, and serotonin can affect mood and how the brain communicates (17). Inflammation in the brain can worsen damage to neurons, while changes in how different brain regions connect can alter how the brain works. At the same time, stress from life challenges can make these problems worse by affecting hormones and the immune system (18).

All these factors interact and create a cycle that leads to and worsens depression after ICH.

### 2.1 Monoamine neurotransmitter imbalance

The neurobiological mechanisms of PICH-D involve complex changes in neurotransmitter systems and neurotrophic factors (19). A major factor is the imbalance of monoamine neurotransmitters, such as serotonin, dopamine, and norepinephrine, due to disrupted pathways in the fronto-striatal circuit, a key brain network for emotional regulation. This imbalance impairs mood control (9). Anatomical evidence reveals that serotonergic (5-HT) fibers in this circuit originate primarily from the raphe nuclei and project to the prefrontal cortex and striatum via the medial forebrain bundle, while noradrenergic (NE) fibers arise from the locus coeruleus and innervate limbic structures through the dorsal bundle (20). Hemorrhagic lesions disrupting these monoaminergic pathways result in dysregulation of the dynamic balance between prefrontal cortical regions (responsible for cognitive control) and limbic structures such as the amygdala and hippocampus (mediating emotional responses), creating a “top-down” regulatory decompensation state.

Clinical investigations demonstrate significantly reduced cerebrospinal fluid levels of 5-hydroxyindoleacetic acid (5-HIAA), the primary 5-HT metabolite, in hemorrhagic stroke patients, with this decrease showing negative correlation with depressive symptom severity (21). This monoaminergic dysfunction may stem from multiple interacting mechanisms including peri-hematomal edema-induced inhibition of tryptophan hydroxylase activity, abnormal presynaptic 5-HT reuptake, and astrocytic glutamate-glutamine cycle disturbances.

Emerging evidence highlights decreased serum Brain-Derived Neurotrophic Factor (BDNF) levels as another pivotal mechanism in post-hemorrhagic depression. As a crucial mediator of neuroplasticity, BDNF deficiency correlates strongly with impaired neuronal functionality (22). Molecular studies elucidate that BDNF exerts its effects through TrkB receptor-mediated activation of the PI3K/Akt signaling pathway, which supports both neurogenesis in hippocampal dentate gyrus and structural plasticity of dendritic spines in prefrontal pyramidal neurons (23). Preclinical findings from striatal hemorrhage models reveal concurrent reductions in prefrontal BDNF expression and dendritic spine density at 7 days post-hemorrhage, with these synaptic remodeling deficits significantly associated with prolonged immobility time in forced swim tests - a behavioral marker of depression (24).

### 2.2 The bidirectional relationship between neuroinflammation and post-intracerebral hemorrhage depression

**Figure 1:**
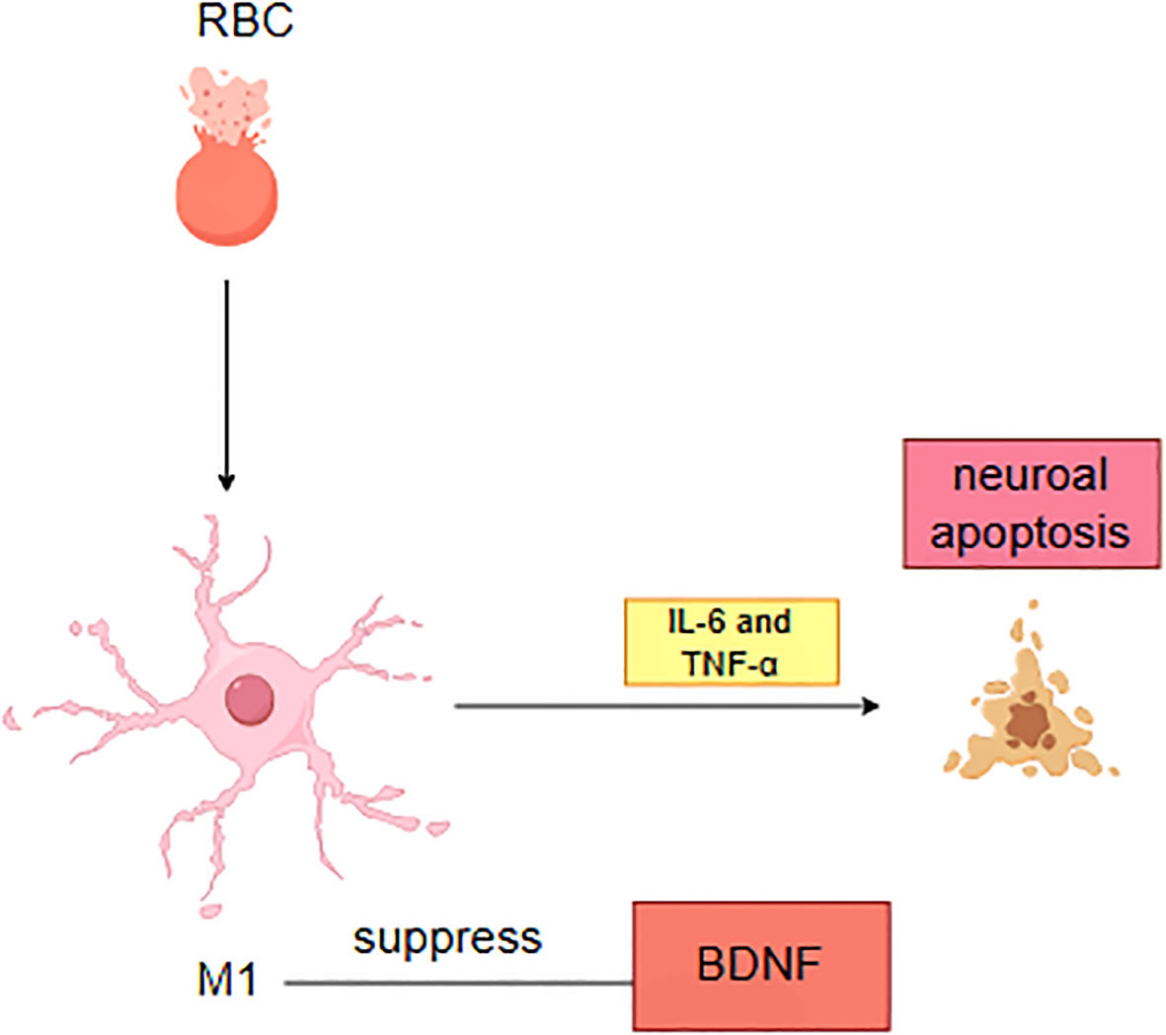
Pathological mechanisms of neuroinflammation and neuronal injury following intracerebral hemorrhage.

Recent investigations have highlighted the reciprocal interactions between neuroinflammatory responses and depression following ICH (25). On one hand, secondary neuroinflammation triggered by ICH serves as a critical pathological foundation for depressive symptoms. During cerebral parenchymal hemorrhage, hemoglobin degradation products (e.g., heme and iron ions) and thrombin activate microglia, driving their polarization toward the pro-inflammatory M1 phenotype (10). Activated M1 microglia subsequently amplify the release of inflammatory mediators, including IL-6 and TNF-α, through the TLR4/NF-κB signaling cascade (26). These cytokines not only induce neuronal apoptosis via activation of caspase-3 pathways but also suppress brain-derived neurotrophic factor (BDNF) synthesis in the hippocampus, impairing neuroplasticity (**Figure 1**).

Conversely, emerging evidence suggests that depressive states may independently amplify neuroinflammatory processes. Elevated systemic inflammatory markers observed in depressed patients create a self-perpetuating cycle, where chronic inflammation exacerbates depressive symptoms and complicates therapeutic interventions (11). Notably, anti-inflammatory strategies such as low-dose minocycline administration demonstrate dual therapeutic potential. By suppressing microglial activation and attenuating pro-inflammatory cytokine release, minocycline effectively mitigates neuroinflammation (27). This neuroprotective effect not only facilitates neural repair but also improves mood regulation, likely through restoration of neuronal function in emotion-related circuits. Such findings underscore the therapeutic value of targeting neuroimmune interactions in ICH-related depression management.

### 2.3 Neuroplastic remodeling and functional aberrations in brain networks

Structural damage caused by cerebral hemorrhage in specific brain regions (e.g., prefrontal cortex, anterior cingulate cortex, basal ganglia) may impair emotional regulation through dual mechanisms: direct disruption of anatomical integrity in cortico-limbic circuits and secondary neuroplastic adaptations driving functional network reorganization (28). Resting-state functional MRI (rs-fMRI) studies reveal critical insights: patients with depression exhibit significant weakening of functional connectivity between posterior components of the default mode network (DMN, particularly the posterior cingulate cortex) and limbic structures (including amygdala and hippocampus). This functional decoupling demonstrates dose-dependent correlations with the severity of anhedonia (29). Notably, DMN-limbic connectivity strength shows potential as a biomarker for predicting antidepressant treatment response, underscoring its pivotal role in pathological mechanisms.

The spatiotemporal evolution of network remodeling displays clinical heterogeneity. During the acute phase (<3 months), localized network efficiency reduction predominates around lesion sites, while chronic stages (>6 months) manifest abnormal increases in whole-brain functional modularity. This hyper-segregated network topology may underlie persistent deficits in executive function and emotional integration (30). Such aberrant connectivity patterns likely disrupt emotional processing and cognitive control mechanisms, contributing to impaired daily functioning and social adaptation. Comprehensive investigation of cerebral network reorganization and functional abnormalities holds substantial implications for elucidating post-hemorrhagic pathophysiological processes and developing targeted therapeutic interventions.

### 2.4 Pathophysiological mechanisms linking oxidative stress and iron dysregulation in post-intracerebral hemorrhage depression

Emerging evidence highlights the critical role of iron metabolism dysregulation and oxidative stress in the pathogenesis of PICH-D. During the acute phase of ICH, hemoglobin degradation releases substantial free iron ions, which catalyze reactive oxygen species (ROS) generation via Fenton reactions (Fe^2+^ + H_2_O_2_ → Fe^3+^+ •OH + OH^−^). This process triggers a cascade of oxidative stress reactions, establishing a neurotoxic microenvironment (31). Concurrent alterations in serum iron-regulatory proteins, particularly elevated ferritin and reduced transferrin levels, demonstrate significant correlations with depressive symptom development (32).

Excessive ROS production induces mitochondrial membrane lipid peroxidation, impairing electron transport chain complexes I-IV function and reducing ATP synthesis efficiency by 40-60%. These metabolic disturbances are exacerbated by mitochondrial DNA damage and cytochrome C release, culminating in neuronal energy crises and apoptotic activation (33). Crucially, systemic iron homeostasis disruption manifests distinct serum biomarkers: hyperferritinemia (>300 ng/mL) indicates iron overload, while decreased transferrin saturation (<20%) reflects impaired iron transport (34). Such systemic dysregulation may permeate the compromised blood-brain barrier, exacerbating iron deposition in limbic structures (e.g., hippocampus and prefrontal cortex). Clinical investigations validate serum ferritin as both a prognostic biomarker and therapeutic target for PICH-D (32).

At the molecular level, oxidative stress impairs neuroplasticity through three primary mechanisms: 1) ROS-mediated suppression of BDNF/TrkB signaling reduces dendritic spine density (35);2) Inhibition of hippocampal neurogenesis (36);and 3) Disruption of glutamate cycling, leading to excitotoxic synaptic accumulation (37). These alterations collectively establish the neurobiological substrate for depressive behaviors. Preclinical studies demonstrate that iron chelation therapy (e.g., desferrioxamine) significantly reduces immobility time in forced swim tests, underscoring its therapeutic potential (38). Emerging therapeutic strategies targeting this axis include precision iron chelation, nano-antioxidant delivery systems, and transferrin receptor modulation. Collectively, interventions addressing iron homeostasis and oxidative damage represent promising avenues for managing PICH-D.

### 2.5 Post-stroke depression: a multidimensional pathogenesis

Psychological and social factors significantly contribute to the pathogenesis of post-stroke depression (PSD). The severity of these symptoms demonstrates a positive correlation with the degree of neurological impairment. Post-acute phase patients frequently experience a self-efficacy crisis secondary to sudden functional limitations (hemiparesis, aphasia, etc.), which profoundly impacts daily living autonomy.

Emerging evidence reveals that chronic psychological stress initiates neurobiological cascades through hypothalamic-pituitary-adrenal (HPA) axis hyperactivity. Preclinical investigations demonstrate that sustained stress exposure downregulates hippocampal glucocorticoid receptor expression, impairing negative feedback inhibition and perpetuating cortisol hypersecretion (39). his neuroendocrine dysregulation directly modulates monoaminergic neurotransmission, particularly enhancing serotonin transporter density in the prefrontal cortex which consequently depletes synaptic 5-hydroxytryptamine (5-HT) availability (40).

The stress-buffering hypothesis elucidates how psychosocial factors modulate post-stress depression susceptibility. The stress-vulnerability model posits that adverse life events (e.g., spousal bereavement, financial crises) exacerbate depression risk by compromising psychological resilience thresholds. Epidemiological data from longitudinal cohorts indicate significantly elevated PSD incidence among individuals experiencing major stressors compared to control populations (41).

Notably, there is a two-way relationship between psychosocial stressors and neurobiological mechanisms, creating a self-sustaining “depression-neurodegeneration” cycle. Modern treatment approaches therefore recommend combining psychosocial rehabilitation with biological therapies to break this harmful cycle effectively.

## 3 Strategy: integrating precision and innovation

The management of PICHD requires a dual focus on neural repair and mood regulation, given its complex causes, including neuroinflammation, monoamine dysregulation, and impaired neural network remodeling. Below, we outline evidence-based interventions targeting these key areas:

### 3.1 Precision therapeutics in pharmacological management

SSRIs, particularly sertraline, remain a first-line pharmacological treatment recommended in current clinical guidelines (e.g., AHA/ASA guidelines) due to their established efficacy and safety profile in stroke populations. A 2019 multicenter randomized controlled trial (RCT) demonstrated that sertraline (50–100 mg/day) significantly reduced Hamilton Depression Rating Scale (HAMD-17) scores after 8 weeks compared to placebo (42). Escitalopram exhibits dual therapeutic benefits through σ1 receptor agonism, with meta-analyses confirming enhanced response rates and concurrent improvement in post-stroke cognitive impairment (43). Clinical protocols mandate thromboelastography monitoring during SSRI administration to mitigate potential coagulation abnormalities, particularly in patients with recent hemorrhage.

Esketamine has emerged as a breakthrough therapy for treatment-resistant cases. Phase III trial results (TRANSFORM-3) revealed that intranasal esketamine combined with oral antidepressants achieved significantly higher remission rates at week 4 compared to placebo plus antidepressants (44). However, its application warrants caution due to transient hemodynamic effects. A recent safety analysis documented associated increases in blood pressure, mandating rigorous cardiovascular monitoring (45).Advanced neuroimaging utilizing resting-state functional magnetic resonance imaging (fMRI) elucidates ketamine’s neuromodulatory effects, particularly enhanced functional connectivity within the default mode network (DMN), which correlates with therapeutic response (46). Notably, current guidelines classify ketamine derivatives as experimental interventions for treatment-resistant depression, restricting their use to specialized settings.

Notably, current guidelines classify ketamine derivatives as experimental interventions for treatment-resistant depression, restricting their use to specialized settings with cardiovascular monitoring due to potential hemodynamic effects.

Minocycline administered at 50 mg twice daily exhibits a dual-pathway therapeutic mechanism for neuroinflammatory modulation, combining microglial activation suppression with synaptic plasticity enhancement (47). Through inhibition of microglial activity, it achieves a significant reduction in pro-inflammatory cytokines IL-6 and TNF-α from baseline level (47), while concurrently upregulating brain-derived neurotrophic factor (BDNF) to higher concentrations compared to placebo (27). This unique immunomodulatory profile, simultaneously addressing neuroinflammatory processes and promoting neurotrophic support, establishes minocycline as a viable adjunctive treatment option for psychiatric disorders associated with neuroimmune dysregulation, particularly those involving both inflammatory pathology and synaptic dysfunction.

### 3.2 Innovative applications of neuromodulation technologies

#### 3.2.1 Neuromodulation therapies

Recent advancements in repetitive transcranial magnetic stimulation (rTMS) demonstrate revolutionary progress through parameter optimization. A recent clinical study demonstrated that low-frequency (1 Hz) rTMS applied to the unaffected motor cortex significantly modulated the kynurenine pathway, a key inflammatory-metabolic cascade implicated in depression, with differential effects based on stimulation laterality (48). These interventions are considered guideline-recommended alternatives when pharmacological treatments are ineffective or poorly tolerated.

#### 3.2.2 Psychotherapy and digital interventions

Cognitive behavioral therapy (CBT) is recommended as a first-line intervention for major depression (American Psychological Association [APA] Level A evidence) and demonstrates particular efficacy in PSD when adapted for neurological deficits (49). A recent RCT demonstrated that CBT combined with sertraline achieved significantly higher response rates compared to pharmacotherapy alone (50). Adaptations for aphasic patients, incorporating visual aids and simplified cognitive restructuring, were critical for feasibility and engagement.

Virtual reality exposure therapy (VRET) has evolved through innovative integration of contextual simulation and biofeedback mechanisms. Modern systems incorporate EEG-neurofeedback synchronization, enabling patients to visually track prefrontal alpha-wave power fluctuations during adaptive training in simulated environments such as virtual supermarkets and social interaction scenarios (51).

### 3.3 The multidisciplinary collaborative approach

The multidisciplinary collaborative approach is implemented through a three-phase intervention model led by neuropsychiatry-focused MDT (Multidisciplinary Team) teams (52). In the acute phase (0-1 month post-onset), neurologists initiate neuroprotective therapies while collaborating with psychiatrists for precise antidepressant titration. During the recovery phase (1-3 months), rehabilitation specialists introduce personalized interventions, such as mirror therapy for post-stroke limb neglect. Transitioning to the maintenance phase (>3 months), psychologists employ mindfulness-based cognitive therapy (MBCT) for relapse prevention. This continuum of care is supported by three operational pillars: weekly standardized MDT case reviews, a cross-departmental intelligent EMR system enabling real-time data sharing, and machine learning models integrating clinical biomarkers for risk prediction. Machine learning models integrating clinical and imaging biomarkers for treatment response prediction, with advanced algorithms such as XGBoost demonstrating superior performance in recent dedicated studies. Specifically, an XGBoost model incorporating clinical indicators (e.g., frontal lobe lesion, NIHSS, PSQI, MMSE) and biochemical markers (e.g., ALB) achieved an AUC of 0.941, accuracy of 87.6%, sensitivity of 82.2%, and specificity of 89.9% in predicting post-stroke depression risk (53). The integrated framework establishes closed-loop management from acute intervention to long-term functional recovery, enhancing therapeutic precision through temporal-stage specialization and multidimensional data synthesis.

## 4 Future research directions and challenges

PICH-D exhibits marked heterogeneity in its pathogenesis, necessitating breakthrough advancements in three key domains: precision medicine frameworks, diagnostic innovation, and interdisciplinary translational research.

Precision medicine uses biomarkers to guide treatment. Studies show that 5-HTTLPR gene differences affect how well SSRIs work. People with the S allele may see slower results and respond less compared to others (54). Furthermore, emerging evidence identifies the combined biomarkers of elevated serum IL-6 levels and reduced BDNF concentrations as reliable predictors of depression severity (55), laying the foundation for multidimensional biomarker assessment systems. These discoveries not only deepen our understanding of PSD’s molecular mechanisms but also pave the way for personalized treatment protocols.

The evolution of multimodal prediction models has progressed from unidimensional analysis to integrative multisource data synthesis. Cutting-edge research reveals that machine learning models integrating amygdala functional connectivity (derived from resting-state fMRI) with serum miR-124 levels achieve superior predictive accuracy for depression risk stratification (46). Such artificial intelligence-enhanced systems enable not only early identification of high-risk populations but also optimization of therapeutic windows through dynamic monitoring of hippocampal volumetric changes. This paradigm shift underscores the transformative potential of computational neuroscience in revolutionizing PSD management.

Target selection for DBS has achieved significant progress in treating psychiatric disorders. A randomized controlled trial demonstrated superior efficacy of bilateral subgenual anterior cingulate cortex (sgACC) stimulation over sham control after 12-week intervention. Concurrently, MRI-guided focused ultrasound (MRgFUS) has emerged as a non-invasive alternative, with preclinical studies showing its capacity to reverse depression-associated neuroplasticity in limbic-thalamo-prefrontal circuits. Clinical trials targeting nucleus accumbens ablation achieved 50% remission rate in treatment-resistant cases (56).

Recent interdisciplinary advances have significantly enhanced our understanding of PICH-D. Mechanistic studies elucidate that gut microbiota-derived tryptophan metabolites modulate serotonergic neuronal activity in the dorsal raphe nucleus via vagal afferent pathways. Recent interdisciplinary research has illuminated the gut-brain axis in post-ICH depression (PICH-D). A combined omics study identified characteristic gut microbiota alterations in PSD patients, including increased Parabacteroides and Staphylococcus and decreased Eubacterium eligens and Prevotella (57). These changes correlated with plasma metabolite disturbances, notably reduced tryptophan and serotonin and elevated cortisol, suggesting intertwined monoamine and HPA axis dysfunction. A combined model of microbial and metabolic markers achieved superior PSD diagnosis (AUC = 0.940), highlighting the value of multimodal biomarkers and supporting roles of vagal and immune pathways.

Clinical translation of these findings demonstrates that adjunctive Bifidobacterium supplementation potentiates SSRI therapeutic efficacy in treatment-resistant cohorts. Concurrently, digital phenotyping innovations utilizing multimodal biosensor integration (Empatica E4, Oura Ring Gen3) enable real-time mood state monitoring. Machine learning algorithms incorporating cardiac autonomic markers and polysomnographic parameters achieve higher sensitivity in predicting depressive relapse 14 ± 3 days prior to clinical manifestation (58).

These breakthroughs underscore the imperative for cross-disciplinary collaboration in neuropsychiatric research. Current challenges center on regulatory harmonization of companion diagnostics and mitigation of algorithmic bias in underrepresented populations. Future directions emphasize convergent innovation across neurogastroenterology, computational psychiatry, and bioelectronic medicine to achieve precision neurotherapeutics.

## 5 Post-stroke depression following cerebral hemorrhage: a path toward precision management

PICH-D represents a complex clinical entity involving multifaceted neurobiological interactions. Its effective management necessitates coordinated multidisciplinary collaboration across neurology, psychiatry, and rehabilitation medicine. Recent advances in precision medicine have enabled the development of individualized therapeutic strategies incorporating neuroimaging biomarkers, genetic profiling, and molecular signatures. The integration of emerging neuromodulation technologies with artificial intelligence platforms promises to revolutionize diagnostic and therapeutic paradigms. This evolving landscape positions post-hemorrhagic depression management at the threshold of an intelligent precision medicine era, potentially optimizing functional recovery and quality of life for affected individuals.
